# Association of atopic diseases with atrial fibrillation risk: A systematic review and meta-analysis

**DOI:** 10.3389/fcvm.2022.877638

**Published:** 2022-08-30

**Authors:** Rong Zeng, Jing Wang, Ziting Liang, Jintao Zhang, Zihan Wang, Changjuan Xu, Liang Dong

**Affiliations:** ^1^Department of Respiratory, Shandong Provincial Qianfoshan Hospital, Cheeloo College of Medicine, Shandong University, Jinan, China; ^2^Department of Respiratory, Shandong Provincial Qianfoshan Hospital, Shandong Institute of Respiratory Diseases, Shandong University, The First Affiliated Hospital of Shandong First Medical University, Jinan, China

**Keywords:** asthma, allergy rhinitis, atopic dermatitis, atrial fibrillation, meta-analysis

## Abstract

**Background:**

Atopic diseases and atrial fibrillation (AF) seem to share an underlying inflammatory pathology. To date, some population-based studies have explored the relationship between the two. We aimed to conduct a meta-analysis to examine the role of atopic condition in AF risk.

**Methods:**

All relevant observational studies in PubMed and EMBASE databases up to November 2021 were searched. In RevMan 5.3, we used random-effects or fixed-effects models to pool the effect sizes of hazard ratio (HR), odds ratio (OR) and their corresponding 95% confidence intervals (95% CI). In addition, I^2^ and Cochran Q test were used to evaluate the heterogeneity.

**Results:**

A total of 2488 records were retrieved. After screening according to the predetermined criteria, 6 cohort studies and 2 case-control studies were included in this meta-analysis. Herein, the meta-analysis of 6 cohort studies suggested that atopic diseases potentially increased the AF risk with the pooled HR of 1.26 (95%CI,1.14–1.39), while the pooled effect size (OR, 1.04; 95%CI,0.74–1.46) of 2 case-control studies was not statistically significant. Based on the types of atopic diseases, further subgroup analyses of 6 cohort studies revealed that asthma, allergic rhinitis, and atopic dermatitis all potentially increased the risk of subsequent AF with the pooled HR of 1.41 (*n* = 4; 95%CI, 1.25–1.58), 1.12 (*n* = 1; 95%CI,1.10–1.14) and 1.06 (*n* = 3; 95%CI, 1.01–1.12), respectively.

**Conclusion:**

This meta-analysis demonstrated that patients with atopic diseases have a higher risk of developing AF, particularly those with asthma.

## Introduction

Atrial fibrillation (AF) is the most common type of cardiac arrhythmia in adults, with an increasing trend in both morbidity and mortality (1). The recognized risk factors related to the development of AF include aging, male sex, obesity, alcohol consumption, smoking, hypertension, diabetes mellitus, obstructive sleep apnea, etc. (1, 2). AF has been reported to affect at least 60 million people worldwide, which is also related to several adverse outcomes, such as heart failure, ischemic stroke, systemic embolism, and cognitive decline (1, 3–5).

Atopic diseases are primarily composed of asthma, allergic rhinitis, and atopic dermatitis, and may coexist in the same individuals. Specifically, allergic rhinitis mainly involves the upper airway and is characterized by nasal congestion, rhinorrhea, sneezing, and nasal itching (6). Asthma is a chronic lower airway inflammatory disease, manifested by cough, wheezing, chest tightness, and shortness of breath (7). Atopic dermatitis, also called atopic eczema, is a chronic recurrent skin disease caused by the dysfunction of skin barrier and characterized by itch and inflammatory eczematous lesions (8).

Emerging evidence suggested that inflammation and immune response are involved in the initiation and maintenance of AF (9). Multiple large population-based studies have shown that autoimmune-mediated diseases, such as vasculitis, inflammatory bowel disease, and psoriasis, can significantly increase AF risk (10–12). Allergy, as an inflammatory response mediated by various immunological pathways, also deserves more attention in patients with AF. However, some observational studies of atopic diseases and AF have presented inconsistent results (13–15). For example, baseline data from a cohort study involving up to 1 million British adults showed asthma was not independently associated with AF (13). Silverwood et al. and Choi et al. noted that atopic patients had a significantly higher AF frequency than non-atopic patients during follow-up (14, 15). Hence, this meta-analysis was performed to determine the relationship between atopic diseases and AF risk in individuals without pre-existing AF.

## Methods

The statement of Preferred Reporting Items for Systematic Reviews and Meta-Analyses (PRISMA) was followed to perform the meta-analysis (16). Besides, we registered the protocol of our meta-analysis in the International prospective register of systematic reviews (PROSPERO, registration number: CRD42022300963).

### Data sources and search strategy

Two investigators (JW, and RZ) independently retrieved relevant articles in PubMed and EMBASE from inception to the search end (November 26, 2021). The search terms were mainly involved with “atrial fibrillation” and “atopic diseases.” We did not restrict language, region of study, or sample size for publications retrieved. The details of our search strategy are summarized in Supplementary Table 1.

### Study selection

Inclusion criteria: (1) observational (cohort or case-control) studies; (2) cohort studies must include patients with atopic diseases and non-atopic individuals. And, the relative risk (RR) or hazard risk ratio (HR) with 95% confidence intervals (95% CI) of incident AF comparing between patients with atopic diseases and individuals without atopic diseases must be reported. (3) case-control studies must consist of patients with AF and controls. Besides, the studies also need to trace their allergic history and provide odds ratio (OR) with 95%CI of the association between atopic diseases and AF. Exclusion criteria: (1) basic experimental researches; (2) lacking or not available data; (3) case reports, reviews, letters, replies, or comments; (4) duplicate publications of the same database.

### Data extraction and quality assessment

For all qualified studies, a standardized collection list was used to extract the following details: first author’s name, publication year, country of the study, study design, the age of participants, sample size, the approaches of disease definition, variables adjusted in multivariate analysis.

Next, the Newcastle-Ottawa Scale (NOS) was used to evaluate the quality of all included studies. For any controversial contents, the three researchers (RZ, JW, and ZTL) discussed and made decisions together. The details of scoring were outlined in Supplementary Table 2.

### Statistical analysis

The effect sizes of HR, OR, and their corresponding 95%CI were extracted as the primary analysis statistics. In addition, based on study design and the types of atopic diseases, we performed subgroup analyses to clarify the association between atopic diseases and the risk of AF. For the heterogeneity of included studies, we assessed it qualitatively and quantitatively using Cochrane Q test and I^2^, respectively. In detail, the value of I^2^ was divided into 4 grades, namely high heterogeneity (>75%), moderate heterogeneity (51–75%), low heterogeneity (26–50%), and insignificant heterogeneity (<25%) (17). During the meta-analysis, the effect model was selected according to the degree of heterogeneity between studies, that is, if the heterogeneity was significant (*I*^2^ > 50%), a random effect model was used to pool the effect size. What’s more, we further used sensitivity analysis to find the sources of high heterogeneity.

In this study, data were analyzed using the software of RevMan 5.3^1^ and STATA 15.0 (Stata Corporation, College Station, TX, United States). Two-sided *p* < 0.05 was viewed as statistically significant.

## Results

### Literature search

**FIGURE 1:**
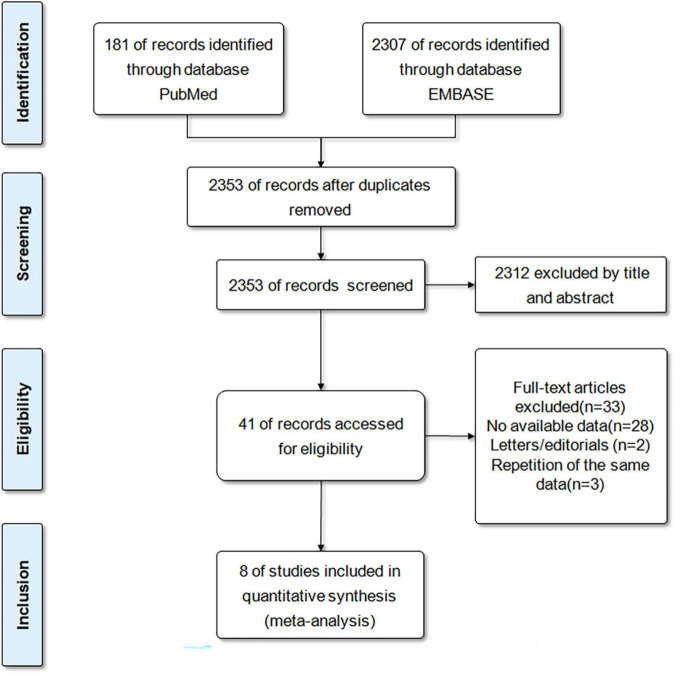
The flow diagram of literature review and study selection.

A total of 2488 records were retrieved from the databases of PubMed and EMBASE. After eliminating duplicates, the 2353 records were reviewed with titles and abstracts. After initial screening, 2312 articles were excluded because they were inconsistent with the scheduled criteria. Then, we conducted a comprehensive review of the full text of 41 articles and eliminated 33 articles because of insufficient results, or repeated publication of the same database. Eventually, a total of 8 studies (6 cohort studies and 2 case-control studies) (14, 15, 18–23) were included for our meta-analysis. Figure 1 demonstrated the selection process.

### Characteristics of included studies

The included six cohort studies consisted of 1,452,112 patients with atopic diseases and 7,373,745 comparators. Three studies were conducted in Europe, 2 in Asia, and 1 in North America. Regarding specific types of atopic diseases, 4 studies investigated the association between asthma and AF, 3 studies examined the relationship between atopic dermatitis and AF, and another study involved allergic rhinitis and AF. All 6 studies extracted the effect size of HR, with 5 providing adjusted HR and 1 providing crude HR.

Among the 2 case-control studies, one from Asia (China), and the other from North America (United States). In total, they traced the history of asthma in 8,869 patients with AF and 11,505 controls, and used the adjusted OR (aOR) to assess the relationship between the two.

Both cohort and case-control studies mainly focused on adult patients, and the definitions of AF and atopic diseases (asthma, allergic rhinitis and atopic dermatitis) were based on matching International Code Designator, documentation of medication, self-reported or physician recorded diagnosis of disease. Moreover, based on the four characteristics: daytime symptoms (2 times per week or less or more than 2 times per week), night awakenings (none or any), the need for reliever medication (2 times per week or less or more than 2 times per week), and limitation of activities (none or any), Cepelis et al. (21) classified asthma as controlled (no above asthma characteristics), partly controlled (2 or less of the above characteristics), and uncontrolled (3 or more of the above characteristics). In another study, Tattersall et al. (23) stratified asthma into two subgroups: persistent asthma (defined as those with asthma on controller medications) and intermittent asthma (those with asthma not taking controller medications). The basic characteristics of the included cohort and case-control studies were shown in Tables 1, 2, respectively.

**TABLE 1:** The baseline characteristics of cohort studies included in the meta-analysis.

Author/year	Date source and country	Study design	Type and number of patients with atopic diseases	Number of comparators	Age	Variables adjusted in multivariate analysis	NOS score
Yang et al. (18)	The Taiwan National Health Insurance database; China	cohort	asthma:21603	86115	≥ 18 years	NA	6
Cepelis et al. (21)	The Nord-Trøndelag Health Study: HUNT2 and HUNT3; Norway	cohort	controlled asthma:2947 partly controlled asthma:1807 uncontrolled asthma:547	48606	≥ 20 years	age, sex, BMI, smoking status, alcohol use, physical activity, education level, waist-to-hip ratio, diabetes mellitus.	8
Silverwood et al. (14)	The UK Clinical Practice Research Datalink; United Kingdom	cohort	atopic eczema:387439	1528477	≥ 18 years	BMI, smoking at cohort entry, time-varying hyperlipidemia, hypertension, depression, anxiety, diabetes, severe alcohol use, current calendar period, time since diagnosis, index of multiple deprivation at cohort entry, time-varying asthma.	9
Schmidt et al. (22)	The Danish National Patient Registry; Denmark	cohort	atopic dermatitis:13126	124211	0-63 years	birth year, sex, index date, chronic obstructive pulmonary disease, cardiovascular disease, rheumatic disease, sleep apnea, hospital-diagnosed obesity, hyperthyroidism, chronic kidney disease, diabetes mellitus, alcohol-related disease, educational level.	8
Tattersall et al. (23)	The Multi-Ethnic Study of Atherosclerosis; United States	cohort	intermittent asthma:497 persistent asthma:150	5968	62.0 ± 10.2 years	age, race, sex, BMI, systolic blood pressure, smoking status, alcohol use, hypertension medication use, diabetes mellitus, education.	8
Choi et al. (15)	The NHIS-National Health Screening Cohort (NHIS-HEALS) database; Korea	cohort	asthma:111874 atopic dermatitis:17073 allergic rhinitis:895049	5580368	40-79 years	age, sex, history of smoking, drinking level, low income, diabetes mellitus, hypertension, dyslipidemia, inflammatory bowel disease, psoriasis.	9

**TABLE 2:** The baseline characteristics of case-control studies included in the meta-analysis.

Author/year	Date source and country	Study design	Number of patients with atrial fibrillation	Number of controls	Age	Variables adjusted in multivariate analysis	NOS score
Chan et al. (19)	The National Health Insurance Research Database in Taiwan; China	case-control	7439	10075	≥ 18 years	age, gender, history of diabetes, hypertension, congestive heart failure, coronary artery disease, chronic renal failure, medications.	7
Chamberlain et al. (20)	The Rochester Epidemiology Project; United States	case-control	1430	1430	≥ 18 years	obesity, hypertension, heart failure, coronary artery disease, chronic kidney disease, chronic obstructive pulmonary disease.	7

### Meta-analysis of cohort studies

**FIGURE 2:**
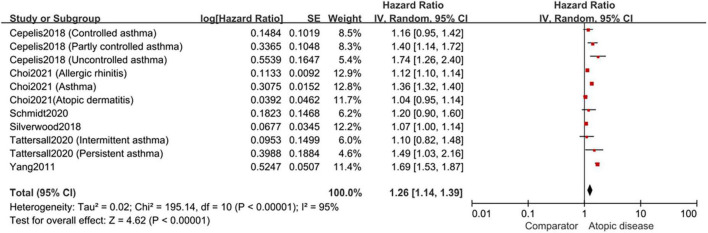
Forest plot depicting the association of atopic diseases with AF risk in cohort studies (HR 1.26, 95% CI 1.14–1.39, *I*^2^ = 95%).

The pooled result of 6 cohort studies indicated that individuals with atopic diseases had a significantly higher risk of AF (HR,1.26; 95%CI,1.14–1.39); nevertheless, the high heterogeneity was observed (*I*^2^ = 95%, *p* < 0.001) (Figure 2).

### Meta-analysis of case-control studies

**FIGURE 3:**
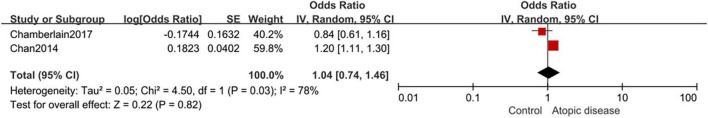
Forest plot depicting the association of atopic diseases with AF risk in case-control studies (OR 1.04, 95% CI 0.74–1.46, *I*^2^ = 78%).

The pooled result of 2 case-control studies was not statistically significant, meaning that individuals with asthma had no increased risk of AF (aOR,1.04; 95%CI,0.74–1.46), and the heterogeneity was also high with I^2^ of 78% (Figure 3).

### Subgroup analyses by the types of atopic diseases

**FIGURE 4:**
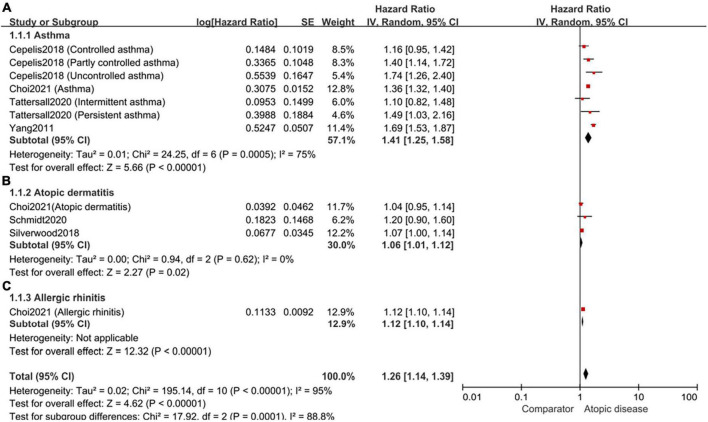
Forest plot of subgroup analyses by the types of atopic diseases. **(A)**: the association of asthma and the risk of subsequent AF (HR 1.41, 95% CI 1.25–1.58, *I*^2^ = 75%); **(B)**: the association of atopic dermatitis and the risk of subsequent AF (HR 1.06, 95% CI 1.01–1.12, *I*^2^ = 0%); **(C)**: the association of allergic rhinitis and the risk of subsequent AF (HR 1.12, 95% CI 1.10–1.14).

Based on the types of atopic diseases, the included cohort studies were divided into three subgroups. Specifically, four studies explored the association between asthma and the risk of AF, with a pooled HR of 1.41 (95%CI, 1.25–1.58; *I*^2^ = 75%) (Figure 4A). For atopic dermatitis, the pooled HR among three studies was 1.06 (95%CI, 1.01–1.12; *I*^2^ = 0%) (Figure 4B). Only one study examined the relationship between allergic rhinitis and the risk of AF, with a HR of 1.12 (95%CI,1.10–1.14) (Figure 4C).

### Subgroup analyses by the severity of atopic dermatitis

**FIGURE 5:**
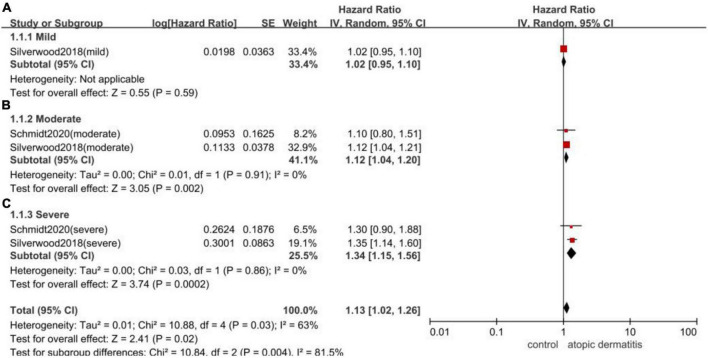
Forest plot of subgroup analyses by the severity of atopic dermatitis. **(A)**: the association of mild patients with atopic dermatitis and the risk of subsequent AF (HR 1.02, 95% CI 0.95–1.10); **(B)**: the association of moderate patients with atopic dermatitis and the risk of subsequent AF (HR 1.12, 95% CI 1.04–1.20, *I*^2^ = 0%); **(C)**: the association of severe patients with atopic dermatitis and the risk of subsequent AF (HR 1.34, 95% CI 1.15–1.56 *I*^2^ = 0%).

Given 2 of the included studies classified patients with atopic dermatitis into mild, moderate and severe groups, the subgroup meta-analysis were performed and indicated that moderate and severe patients had a higher risk of AF, with the HR of 1.12 (95%CI,1.04–1.20; *I*^2^ = 0%) and 1.34 (95%CI,1.15–1.56; *I*^2^ = 0%), respectively (Figure 5).

### Sensitivity analysis

**FIGURE 6:**
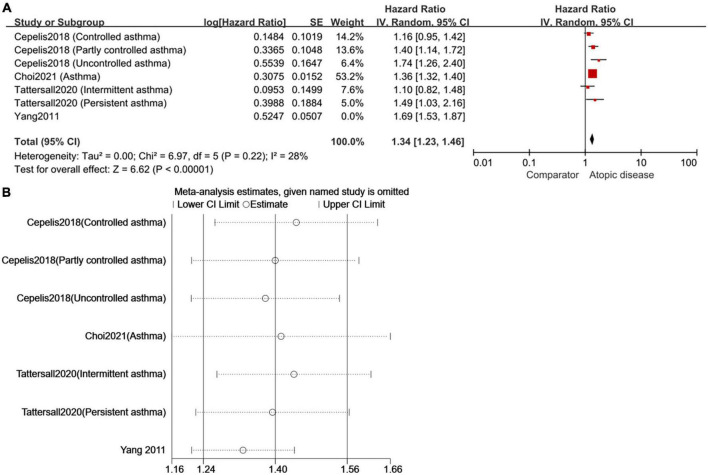
To repeat the sensitivity analysis, the image used two types of software [**(A)** RevMan 5.3 and **(B)** STATA 15.0]. As expected, the results were consistent and suggested that Yang et al. (18) was the main factor of high heterogeneity in the meta-analysis of cohort studies on AF risk in asthmatic patients.

Despite the subgroup analyses, high heterogeneity still existed in studies involving the relationship between asthma and the risk of AF. Thus, we conducted sensitivity analysis by omitting the study one by one and found Yang et al. 2011 (18) was the main influencing factor of high heterogeneity. In detail, heterogeneity was reduced from 75 to 28% when Yang et al. 2011 (18) was removed from the subgroup about the relationship of asthma and AF. Meanwhile, the pooled HR changed from 1.41 (95%CI, 1.25–1.58) to 1.34 (95%CI, 1.23–1.46) (Figure 6).

### Publication bias

Given the limited number of included studies, we assessed publication bias in the subgroups of cohort studies and asthma cohort studies. There was no obvious publication bias in either subgroup (funnel plots in Supplementary Figures 1, 2 were roughly symmetrical, and both Egger’s and Begg’s tests presented that *p* > 0.1).

## Discussion

To our knowledge, this meta-analysis concluded the results of all qualified cohort and case-control studies and examined the association between atopic diseases and the risk of AF for the first time. Overall, based on cohort studies, the meta-analysis demonstrates a high risk of developing AF among atopic patients, although the pooled result of two case-control studies is not statistically significant. Further, in terms of the types of atopic diseases, patients with asthma are at the greatest risk for AF development (the HR is 1.41), especially those in persistent or uncontrolled condition. Allergic rhinitis and atopic dermatitis were second to asthma in their association with the risk of new-onset AF (the HR is 1.12 and 1.06 for allergic rhinitis and atopic dermatitis, respectively).

Currently, the potential mechanism that could explain the association between atopic diseases and AF may be chronic systemic inflammation. As we all know, after exposure to environmental allergens, epithelial-derived alarmins (Interleukin-25, Interleukin-33 and thymic stromal lymphopoietin-TSLP) could activate T helper type 2 (Th2) cells and group 2 innate lymphoid cells (ILC2s). Then these immune cells would release a series of cytokines, such as IL-4, IL-5, and IL-13, which promote the production of immunoglobulin E (IgE), eosinophilic inflammation (24). In addition, studies found that Th1, Th17 cells, and neutrophils were also involved in the pathogenesis of atopic diseases (25–27). IL-6, a pro-inflammatory cytokine positively associated with atopic diseases, has been proven to promote Th2 and Th17 differentiation either alone or together with transforming growth factor-β (TGF-β) (28). Meanwhile, the mediators of IL-6, tumor necrosis factor-α (TNF-α), myeloperoxidase, and pro-matrix metalloproteinase that released from polymorphonuclear neutrophil can stimulate cardiac fibroblasts via the Janus kinase/signal transducers and activators of transcription (Jak/STAT) pathway and activation of MAP kinases, nuclear factor-κB (NF-κB) and others (29). Subsequently, fibroblasts differentiate into myofibroblasts, which produce growth factors such as TGF-β, cytokines such as matrix metalloproteinases and extracellular matrix proteins. Ultimately, the sustained action of the above mediators leads to atrial structural remodeling and the development of AF (29). Indeed, circulating inflammatory biomarkers of C-reactive protein, TNF-α, and IL-6 were also observed to be obviously elevated and related with AF status (i.e., patients with permanent AF have higher levels of inflammatory biomarkers) (30).

Secondly, mast cells, a key regulator of allergy and inflammation, are also abundant in the human heart (31). When allergens are repeatedly exposed to atopic patients, mast cells throughout the body can be activated. Mediators released from activated heart mast cells, such as histamine, leukotrienes, and platelet-activating factors, may directly induce arrhythmias, even sudden cardiac death (31). Moreover, cardiac mast cells seem to play a vital role in the pathogenesis of cardiac fibrosis. In an animal study, infiltration of myocardial mast cells was observed in pressure-overloaded mouse hearts and could cause AF through platelet-derived growth factor A-mediated fibrosis (32).

In this meta-analysis, asthmatics were identified as having a higher risk of developing AF. Due to the different definitions of asthma subtypes, we did not perform relevant subgroup analyses. But it is worth noting that Cepelis et al. (21) found a positive dose-response association between levels of asthma control and AF risk. In addition, Tattersall et al. (23) indicated that persistent asthma, not intermittent asthma, is associated with an increased AF risk. On the one hand, despite active asthma treatment, uncontrolled or persistent asthmatics had higher level of systemic inflammation. What’s more, the stratified analysis based on self-assessed medication use at baseline in asthmatics showed that patients currently treated with inhaled corticosteroids, especially new users, had higher risk than non-users for AF (19). The researchers found that the use of β2-agonists also increased the risk of AF especially in patients with uncontrolled and active asthma (21, 33). As we all know, β2-agonists may stimulate and increase cardiac sympathetic activity and affect the electrophysiological mechanisms of AF initiation and/or maintenance.

The relationship between atopic dermatitis and cardiovascular disease is controversial (34). Our meta-analysis of three studies provides positive evidence for atopic dermatitis and AF risk, although the absolute risk is low. Apart from inflammation, poor health behaviors including smoking and physical inactivity caused by reduced quality of life and psychological stress of atopic dermatitis patients may also contribute to the risk of AF (35). However, as for allergic rhinitis and AF risk, only one study focused on this, and the data also showed a positive association between the two. Last but not least, as we know, atopic march refers to the progress of atopic dermatitis in infancy and subsequent allergic rhinitis and asthma in later childhood (36). In terms of allergens, multiple molecular components from animals and fungi have also been found to be simultaneously elevated in patients suffering from severe form of atopic dermatitis, asthma and allergic rhinitis (37, 38). Therefore, the co-existence of two or even three allergic diseases is not uncommon, and a cohort study of up to 6 million Korean subjects presented evidence that patients with multiple atopic conditions have a higher risk for AF than one alone (15).

### Limitations of study

There are some limitations should be considered when the results of our meta-analysis were interpreted. First, the small number of studies available for inclusion limited the possibility of conducting more subgroup analyses to some extent. Second, the definition of allergic diseases and AF lacks preciseness and specificity. For instance, all the included studies did not distinguish between paroxysmal and persistent AF, so we cannot make a further clinical interpretation. Third, the medication of patients with atopic diseases is a factor that must be considered for their secondary diseases. However, only 2 studies (one cohort study and one case-control study) performed subgroup analyses of the association between medications (β-agonist and corticosteroid) for asthma control and AF risk and demonstrated the positive relationship between the two. In the future, it is necessary to obtain relevant clinical information to clarify the causes of AF in patients with atopic diseases. Besides, the study of Yang et al. 2011 (18) that had a large impact on the heterogeneity of the asthma subgroup was excluded through sensitivity analysis to make the results more reliable. We found Yang et al., 2011 (18), a conference abstract, didn’t provide adjusted HR, which may be the leading cause for increasing inter-study heterogeneity.

## Conclusion

In summary, three atopic diseases (asthma, allergic rhinitis, atopic dermatitis) were noted to increase the risk of AF. In the future, more high-quality cohort and case-control studies should be conducted to confirm the association between atopic diseases and AF, with particular attention to whether the increased risk of different types of AF is related to the type, severity, and treatment of atopic diseases.

## Data availability statement

The original contributions presented in this study are included in the article/Supplementary material, further inquiries can be directed to the corresponding author.

## Author contributions

RZ, JW, JZ, and LD proposed and designed the study. RZ, JW, and ZL contributed to the data collection, statistical analysis, and quality assessment. RZ and JW interpreted the data and wrote the original manuscript of the study. ZL, JZ, ZW, and CX conducted major revisions. All authors critically revised important intellectual content of the study and approved the final version for publication.

## Footnotes

1
www.cochrane.org/

## Supplementary material

Supplementary Figure 1 Evaluation of publication bias in the subgroup of cohort studies.

Supplementary Figure 2 Evaluation of publication bias in the subgroup of asthma cohort studies.
