# Supplementary figures and images for: Association of atopic diseases with atrial fibrillation risk: A systematic review and meta-analysis
Source: Front Cardiovasc Med. 2022 Aug 30;9:877638. doi: 10.3389/fcvm.2022.877638 (PMC9468366; doi:10.3389/fcvm.2022.877638)

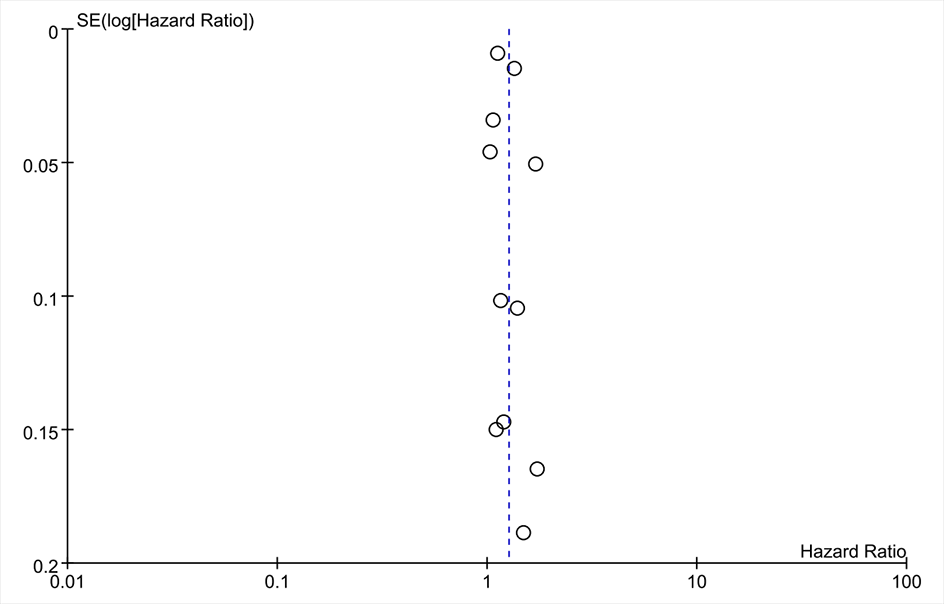

Supplement: Supplementary Figure 1 — Evaluation of publication bias in the subgroup of cohort studies. [file Image_1.TIF]

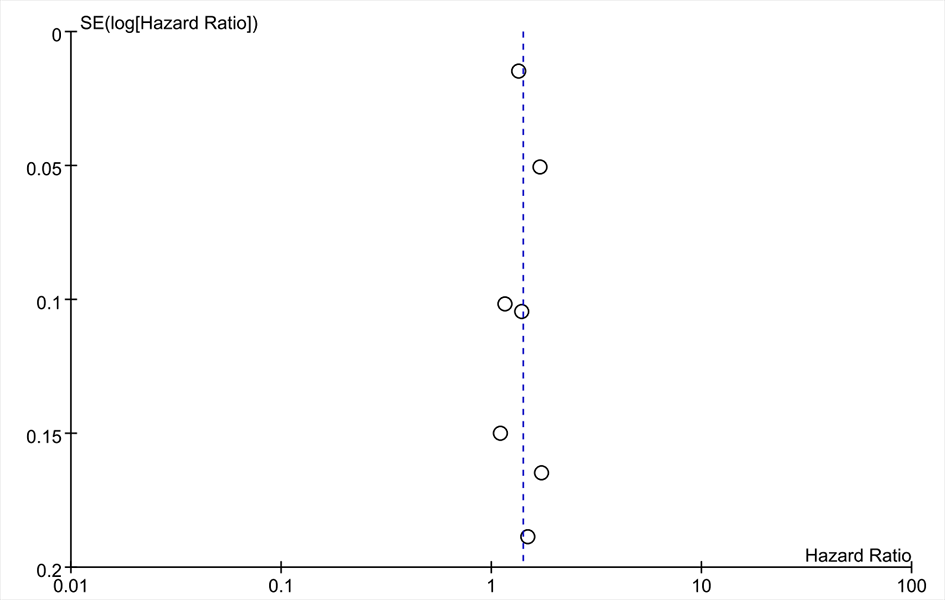

Supplement: Supplementary Figure 2 — Evaluation of publication bias in the subgroup of asthma cohort studies. [file Image_2.TIF]
