# Supplementary material for: Association of atopic diseases with atrial fibrillation risk: A systematic review and meta-analysis
Source: Front Cardiovasc Med. 2022 Aug 30;9:877638. doi: 10.3389/fcvm.2022.877638 (PMC9468366; doi:10.3389/fcvm.2022.877638)
Supplement: Supplementary file 3 [file Table_1.DOC]

Supplementary Table 1. Search strategy used to identify studies

| PubMed |
| --- |
| ((((((((((((((((((((((((((Atrial Fibrillation[MeSH Terms]) OR (Atrial Fibrillations[all fields])) OR (Fibrillation, Atrial[all fields])) OR (Fibrillations, Atrial[all fields])) OR (Auricular Fibrillation[all fields])) OR (Auricular Fibrillations[all fields])) OR (Fibrillation, Auricular[all fields])) OR (Fibrillations, Auricular[all fields])) OR (Persistent Atrial Fibrillation[all fields])) OR (Atrial Fibrillation, Persistent[all fields])) OR (Atrial Fibrillations, Persistent[all fields])) OR (Fibrillation, Persistent Atrial[all fields])) OR (Fibrillations, Persistent Atrial[all fields])) OR (Persistent Atrial Fibrillations[all fields])) OR (Familial Atrial Fibrillation[all fields])) OR (Atrial Fibrillation, Familial[all fields])) OR (Atrial Fibrillations, Familial[all fields])) OR (Familial Atrial Fibrillations[all fields])) OR (Fibrillation, Familial Atrial[all fields])) OR (Fibrillations, Familial Atrial[all fields])) OR (Paroxysmal Atrial Fibrillation[all fields])) OR (Atrial Fibrillation, Paroxysmal[all fields])) OR (Atrial Fibrillations, Paroxysmal[all fields])) OR (Fibrillation, Paroxysmal Atrial[all fields])) OR (Fibrillations, Paroxysmal Atrial[all fields])) OR (Paroxysmal Atrial Fibrillations[all fields])) AND ((((((((Asthma[MeSH Terms]) OR (Asthmas[all fields])) OR (Bronchial Asthma[all fields])) OR (Asthma, Bronchial[all fields])) OR ((((Rhinitis, Allergic[MeSH Terms]) OR (Allergic Rhinitides[all fields])) OR (Rhinitides, Allergic[all fields])) OR (Allergic Rhinitis[all fields]))) OR ((((((((((((((((Dermatitis, Atopic[MeSH Terms]) OR (Atopic Dermatitides[all fields])) OR (Atopic Dermatitis[all fields])) OR (Dermatitides, Atopic[all fields])) OR (Neurodermatitis, Atopic[all fields])) OR (Atopic Neurodermatitides[all fields])) OR (Atopic Neurodermatitis[all fields])) OR (Neurodermatitides, Atopic[all fields])) OR (Neurodermatitis, Disseminated[all fields])) OR (Disseminated Neurodermatitides[all fields])) OR (Disseminated Neurodermatitis[all fields])) OR (Neurodermatitides, Disseminated[all fields])) OR (Eczema, Atopic[all fields])) OR (Atopic Eczema[all fields])) OR (Eczema, Infantile[all fields])) OR (Infantile Eczema[all fields]))) OR ((((((((((((((((((((((Conjunctivitis, Allergic[MeSH Terms]) OR (Conjunctivitis, Atopic[all fields])) OR (Atopic Conjunctivitides[all fields])) OR (Atopic Conjunctivitis[all fields])) OR (Conjunctivitides, Atopic[all fields])) OR (Allergic Conjunctivitis[all fields])) OR (Allergic Conjunctivitides[all fields])) OR (Conjunctivitides, Allergic[all fields])) OR (Conjunctivitis, Vernal[all fields])) OR (Conjunctivitides, Vernal[all fields])) OR (Vernal Conjunctivitides[all fields])) OR (Vernal Conjunctivitis[all fields])) OR (Keratoconjunctivitis, Vernal[all fields])) OR (Keratoconjunctivitides, Vernal[all fields])) OR (Vernal Keratoconjunctivitides[all fields])) OR (Vernal Keratoconjunctivitides[all fields])) OR (Conjunctivitis, Giant Papillary[all fields])) OR (Conjunctivitides, Giant Papillary[all fields])) OR (Giant Papillary Conjunctivitides[all fields])) OR (Giant Papillary Conjunctivitis[all fields])) OR (Papillary Conjunctivitides, Giant[all fields])) OR (Papillary Conjunctivitis, Giant[all fields]))) OR ((allerg*) OR (atop*))) |
| **EMBASE** |
| #1. 'atrial fibrillation'/exp  #2. 'asthma'/exp  #3. 'rhinitis'/exp  #4. 'allergic conjunctivitis'/exp  #5. 'atopic dermatitis'/exp  #5. 'eczema'/exp  #7. allerg*  #8.  atop*  #9. #2 OR #3 OR #4 OR #5 OR #6 OR #7OR #8  #10. #1 AND #9 |
