# Supplementary material for: Association of atopic diseases with atrial fibrillation risk: A systematic review and meta-analysis
Source: Front Cardiovasc Med. 2022 Aug 30;9:877638. doi: 10.3389/fcvm.2022.877638 (PMC9468366; doi:10.3389/fcvm.2022.877638)
Supplement: Supplementary file 4 [file Table_2.DOC]

| Author,year | Selection | | | | Comparability | Outcome | | | Total  Score |
| --- | --- | --- | --- | --- | --- | --- | --- | --- | --- |
|  | Representativeness of the exposed cohort | Selection of the non exposed cohort | Ascertainment  of exposure | Demonstration  that outcome of interest was not  present at start of study | Comparability of cohorts  on the basis of the design or analysis | Assessment  of outcome | Was follow-up long enough for  outcomes to occur? | Adequacy of follow up of cohorts |  |
| Cepelis 2018 | ★ | ★ | ☆ | ★ | ★★ | ★ | ★ | ★ | 8 |
| Schmidt 2019 | ★ | ☆ | ★ | ★ | ★★ | ★ | ★ | ★ | 8 |
| Choi 2021 | ★ | ★ | ★ | ★ | ★★ | ★ | ★ | ★ | 9 |
| Tattersall 2020 | ★ | ★ | ☆ | ★ | ★★ | ★ | ★ | ★ | 8 |
| Silverwood 2018 | ★ | ★ | ★ | ★ | ★★ | ★ | ★ | ★ | 9 |
| Yang 2011 | ★ | ★ | ☆ | ★ | ★☆ | ☆ | ★ | ★ | 6 |

Supplementary Table 2.1 Detailed risk of bias in cohort studies based on the Newcastle Ottawa Scale

**Supplementary Table 2.2 Detailed risk of bias in case-control studies based on the Newcastle Ottawa Scale**

| Author, year | Selection | | | | Comparability | Exposure | | | Total  Score |
| --- | --- | --- | --- | --- | --- | --- | --- | --- | --- |
|  | Is the case definition adequate? | Representativeness of the cases | Selection of controls | Definition of controls | Comparability of cases and controls  on the basis of the design or analysis | Assessment  Of exposure | Same method of ascertainment for cases and controls | No-response rate |  |
| Chan 2014 | ★ | ☆ | ☆ | ★ | ★★ | ★ | ★ | ★ | 7 |
| Chamberlain 2016 | ★ | ☆ | ★ | ★ | ★★ | ★ | ☆ | ★ | 7 |
